# High-Accuracy Self-Calibration for Smart, Optical Orbiting Payloads Integrated with Attitude and Position Determination

**DOI:** 10.3390/s16081176

**Published:** 2016-07-27

**Authors:** Jin Li, Fei Xing, Daping Chu, Zilong Liu

**Affiliations:** 1 Department of Precision Instrument, Tsinghua University, Beijing 100084, China; jl918@cam.ac.uk (J.L.); liuzl@nim.ac.cn (Z.L.); 2 State Key Laboratory of Precision Measurement Technology and Instruments, Beijing 100084, China; 3 Collaborative Innovation Center for Micro/Nano Fabrication, Device and System, Beijing 100084, China; 4 Photonics and Sensors Group, Department of Engineering, University of Cambridge, 9 JJ Thomson Avenue, Cambridge CB3 0FA, UK

**Keywords:** optical orbiting payload, self-calibration, position determination

## Abstract

A high-accuracy space smart payload integrated with attitude and position (SSPIAP) is a new type of optical remote sensor that can autonomously complete image positioning. Inner orientation parameters (IOPs) are a prerequisite for image position determination of an SSPIAP. The calibration of IOPs significantly influences the precision of image position determination of SSPIAPs. IOPs can be precisely measured and calibrated in a laboratory. However, they may drift to a significant degree because of vibrations during complicated launches and on-orbit functioning. Therefore, laboratory calibration methods are not suitable for on-orbit functioning. We propose an on-orbit self-calibration method for SSPIAPs. Our method is based on an auto-collimating dichroic filter combined with a micro-electro-mechanical system (MEMS) point-source focal plane. A MEMS procedure is used to manufacture a light transceiver focal plane, which integrates with point light sources and a complementary metal oxide semiconductor (CMOS) sensor. A dichroic filter is used to fabricate an auto-collimation light reflection element. The dichroic filter and the MEMS point light sources focal plane are integrated into an SSPIAP so it can perform integrated self-calibration. Experiments show that our method can achieve micrometer-level precision, which is good enough to complete real-time calibration without temporal or spatial limitations.

## 1. Introduction

High-resolution Earth observation applications have become essential in many fields, such as mapping, environmental monitoring, and exploration for resources. High-resolution images and high-accuracy image positioning determinations play an important role in Earth observation applications [1,2]. High-accuracy and high-resolution optical imaging payloads have strong requirements on their attitude control precision, orientation, and attitude transfer matrix [3,4]. However, traditional satellite payloads struggle to meet these requirements because traditional satellites mainly use the separation model of platforms and payloads. Platform precisions cannot fulfill the requirements of high-precision imaging payloads. Recently, a high-accuracy space smart payload system integrated with attitude and position (SSPIAP) was developed to meet the requirements for high-resolution and high-accuracy applications [5,6,7,8,9]. The SSPIAP integrates a high-resolution remote camera and miniature attitude-sensitive and position-sensitive devices, such as a star tracker, a micro-electro-mechanical system (MEMS) gyroscope, and a global positioning system (GPS) into a new smart payload system. The SSPIAP can autonomously achieve lightweight and high-performance attitude and position determination with a combination of celestial navigation, inertial navigation, and satellite navigation. Using high-accuracy attitude and position information, the SSPIAP can complete real-time imaging strategy adjustments, imaging, optimal high-resolution remote imaging, high-accuracy image position determination, and so on. Image position determination is one of the most significant functions of an SSPIAP when it is working on satellites. The inner orientation parameters (IOPs) of the SSPIAP, such as principal distance and principal point, strongly influence the accuracy of image position determination. Therefore, calibrations of the IOPs of the SSPIAP are necessary.

On the ground, many approaches to calibrate the IOPs of space optical cameras exist [10,11,12,13,14]. In [15], Yilmazturk used color targets to calibrate color digital cameras. In [16], Ricolfe-Viala et al. used a set of optimal conditions to improve calibration accuracy. In [17], Simon et al. used crossed-phase diffractive optical elements (DOEs) to generate equally spaced dots for wide-angle geometric camera calibration. The DOEs can generate multiple two dimensional (2D) diffraction grids which can be used to calibrate cameras for photogrammetry. In [18], the IOPs were calculated based on the three dimensional (3D) coordinates of several given points and image points. They adopt a four step method using multiple views to solve all the intrinsic parameters. This method can calibrate intrinsic parameters, distortion, and image deformation. The IOPs can be precisely calibrated in a laboratory. However, they may drift to a great degree because of vibration during complicated launches and on-orbit working conditions. The laboratory calibration approaches generally need a calibrated reference object. Thus, these methods are unsuitable for the on-orbit calibration of an SSPIAP.

Recently, several self-calibration methods, that do not require a calibrated reference object, have been developed to calculate IOPs [19,20]. These methods use constraints among the system parameters to calibrate cameras. Self-calibration methods can make it possible to use unknown scenes and motions to calibrate a camera [21]. In [22], Song proposed an active-vision-based self-calibration method. In [23], Caprile et al. performed self-calibration based on vanishing points or lines. In [24], Gonzalez-Aguilera used an iterative, and robust, least squares method to calculate internal calibration parameters combined with a geo-reference, terrestrial laser scanner (TLS) dataset. However, these self-calibration methods have computational complexity, heavy computation, and nontrivial solutions of equations. Meanwhile, calibration accuracy cannot be guaranteed and its robustness is very low. A self-calibrating bundle adjustment is ideal for camera calibrations, for a number of reasons summarized in [25]. In [26], Lichti et al. compared three geometric self-calibration methods for range cameras. The self-calibration bundle adjustment was found to be slightly superior. However, the self-calibrating bundle adjustment method suffers from long computation times [27]. Owing to the limited on-board computer of the SSPIAP platform, these methods are not suitable for high-accuracy calibration of remote sensing SSPIAP applications.

For on-orbit calibration of remote sensing cameras, traditional methods basically use ground control point (GCP) methods [28]. In [29], Fourest et al. adopted stars as control points (CPs) to complete calibration. This method performs calibration during on-orbit commissioning, with numerous measurements made on different stars as seen right around the World. This method adopts a wide base of star CPs. However, the availability and access to GCPs or star CPs is not always easy. Further, the accuracy of each GCP or star CP must be consistent with the requirements of the location system performance.

Recently, 180-degree satellite maneuvers have been used to calibrate on-orbit IOPs [30]. This method needs a standard ground calibration field. In [31], Delvit et al. proposed an auto-reverse method during the commissioning phase. Although this method is efficient and does not require external reference data, its operational implementation is highly constraining because the acquisition of the same site image pair wastes a long orbit portion to complete the alignment to the ground projection of the scan-line on the ground velocity. Therefore, existing on-ground methods and on-orbit methods both possess common characteristics with the aid of external targets. These methods are limited by space and time.

In this paper, we propose an on-orbit, auto-collimating self-calibration method for SSPIAPs. Our method includes four steps. In the first and second steps, a dichroic filter and a MEMS point light sources focal plane are integrated into an SSPIAP to perform the calibration of the IOPs. In the third step, an integration mathematical calibration model is built based on geometric imaging relationships between the first two steps. In the fourth step, a centroid extraction algorithm processes images to extract the star point position. Finally, the principal distance and the principal point can be calculated based on the mathematical calibration model. The SSPIAP can perform integrated self-calibration without temporal and spatial limitations. The rest of this paper is organized as follows: the principle of the proposed method is introduced in Section 2, while, Section 3 describes the experimental results.

## 2. Proposed Method for On-Orbit, Integrated Self-Calibration

### 2.1. Principles

Position determination (positioning) without using ground control points (GCPs) is one of the recent key technical problems for remote sensing photogrammetry. The IKonos-2 satellite can reach positioning accuracy of 15 m without GCPs [32]. The WorldView-2 satellite can reach positioning accuracy of 6.5 m without GCPs [33]. The Geoeye satellite can provide positioning accuracy of 4 m without GCPs [5]. An SSPIAP also adopts a positioning method without GCPs and requires a positioning accuracy of 5 m [6]. An SSPIAP uses forward-looking and back-looking images to complete the digital mapping. This method requires an SSPIAP to have accurate geometrical performance. In order to extract highly accurate topographical information from two overlapping strip images, an SSPIAP must provide highly accurate IOPs. After an adjustment of the optical camera, the IOPs deviate from the ideal values specified by the design, manufacture, assembly, etc. [34,35,36]. Therefore, accurate calibrations of IOPs are necessary for an SSPIAP. For an SSPIAP, positioning accuracy (without GCPs) mainly depends on the accuracy of satellite station positioning, attitude measurement accuracy, image points’ measurement accuracy, IOP measurement accuracy, etc. In order for an SSPIAP to reach a positioning accuracy of 5 m, the distribution of primary errors of the SSPIAP should be as follows: (1) the attitude determination accuracy is within 10″ (angular seconds); (2) the precision orbit determination is within 0.2 m; (3) the angle calibration accuracy between the star tracker and the optical camera is within 5″; (4) camera lens distortion calibration accuracy is within 5 μm; (5) the principal distance calibration accuracy is within 50 μm; (6) the principal point calibration accuracy is within a third of a pixel. After the adjustment of our SSPIAP, we calibrate all of the above parameters. In this SSPIAP, its optical camera uses a design that integrates with the installation structure of the Pico Star Tracker. They have a common benchmark. The Pico Star Tracker is a new type of self-developed, attitude measurement device with 7″ accuracy, which can obtain high accuracy outside the azimuth elements of our SSPIAP and meet the 10″ accuracy requirements. This SSPIAP can, in real-time, attain the benchmarks for the unified, on-orbit, inside-azimuth elements and the angle between the camera optical axis and the star sensor. The optical system of the Pico Star Tracker adopts a transmission structure. Its optical axis is not easily changed. Therefore, the optical axis of the Pico Star Tracker can be replaced by an external mechanical benchmark. In the SSPIAP, the mechanical benchmark of the Pico Star Tracker is similar to the installation structure of the optical camera. Therefore, the angle between the camera optical axis and the star sensor benchmark can also be calibrated by the proposed method. This can ensure that the angle calibration accuracy satisfies the 5″ requirements needed when the SSPIAP is working on a satellite. Usually, the angle variation between the optical axis of the camera and the star sensor benchmark is relatively small.

In this paper, we mainly discuss the on-orbit method of monitoring the principal distance and the principal point when the SSPIAP is working on a satellite. We introduce the calibration of IOPs, including the principal distance and the principal point. We propose an integrated self-calibration method which can be used on-ground and on-orbit.

**Figure 1:**
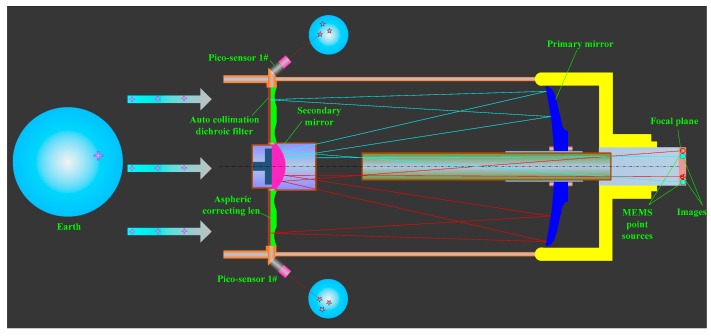
Principle of auto-collimating calibration method.

An auto-collimation dichroic filter and MEMS point sources are integrated into the SSPIAP to perform the calibration of the IOPs. The principle of the proposed, auto-collimating calibration of the IOPs for the SSPIAP is shown in Figure 1. The optical camera system of the SSPIAP includes a primary mirror, a secondary mirror, an aspheric corrector, and a focal plane. The auto-collimation dichroic filter is plated on the plane of the aspheric corrector. This is done to fabricate an auto-collimation light reflection element. The point sources and the image sensor are integrated into the focal plane assembly. The auto-collimating light path includes point sources, a camera lens, an auto-collimation dichroic filter, and an image detector. According to the optical path of the camera, the MEMS point light sources are auto-collimating lights when they pass through the secondary mirror, primary mirror, and the aspheric corrector of camera lenses. The auto-collimating lights are reflected by the auto-collimation dichroic filter and then return into the camera lens. Lights going out of the camera are incident on the focal-plane detector.

In the proposed method, lighting elements and a detection module are introduced into the optical system of the camera between two interleaving focal-plane assemblies. A dichroic filter is also integrated into the optical system of the camera. The proposed method can be summarized in four steps.

**Figure 2:**
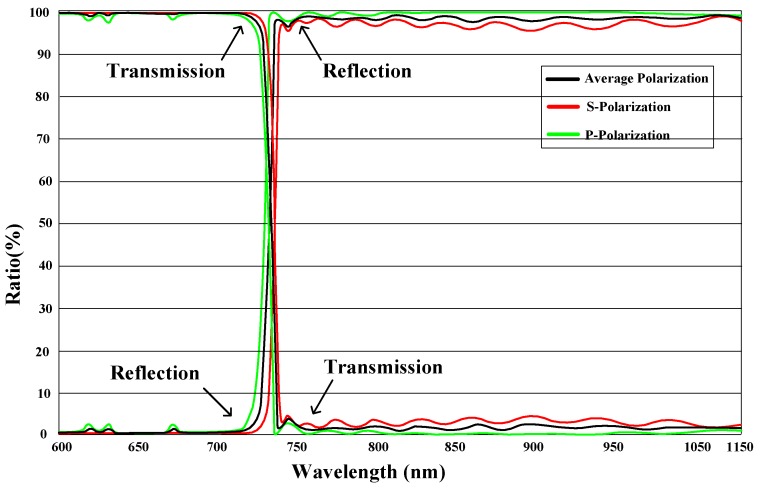
Filtering performance of dichroic filter.

In the first step, an auto-collimation dichroic filter is used to complete the integrated imaging and calibration. The dichroic filter can selectively pass light in a small range of bands while reflecting light in other bands. Figure 2 shows the reflection and transmission ratios of the dichroic filter for the visible optical camera. 

The dichroic filter allows visible light to pass through while reflecting longer wavelength bands. According to the details of the dichroic filter, the bands of MEMS point sources can be determined. When an SSPIAP is working on a satellite, light reflected from and radiated off a target passes through the dichroic filter to complete on-orbit imaging. The lights of the MEMS point sources are reflected by the dichroic filter to complete on-orbit calibration.

**Figure 3:**
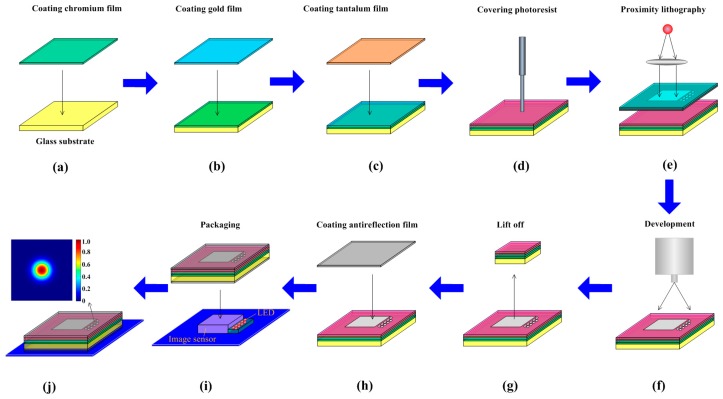
MEMS process to fabricate point-source focal plane.

In the second step, we fabricate a MEMS point source focal plane and then integrate it into the optical focal plane of the SSPIAP. The MEMS point sources are installed on the focal plane, which means that the monitoring optical path is auto-collimating. Thus, the principal distance and the principal point can be monitored when they are needed. To ensure a sufficiently small size and low power consumption, we used MEMS procedures to fabricate a point-source focal plane in this study. The point-source focal plane is mainly composed of the mask that is fabricated by utilizing the MEMS process, a housing, and an electrical system. The assembly of the point-source focal plane is shown in Figure 3.

The fabrication process of the mask is as follows: (1) chromium, gold and tantalum materials are plated on a specified glass substrate; and (2) the photoetching process is performed on the metal layers to obtain several small apertures. The mask includes a glass substrate, a mask layer, and an anti-reflective layer, etc., and the mask thickness is 1.6 mm. The etched apertures in the mask can pass through light, while other parts cannot because they are covered by metal layers. Optical anti-radiation quartz glass is used as the substrate because it can block free space, background cosmic radiation. First, a layer of chromium is plated on the glass substrate. The layer of chromium can completely attenuate the light with its thickness of 75 nm, which depends on the transmissivity of the optical system; Second, the gold membrane is plated on the chromium layer. The gold membrane is a mask layer and its thickness is 200 nm; Third, the tantalum membrane is plated on the gold layer, which is a radiation protection layer, and its thickness is 60 nm; Fourth, the photoresist is poured onto the plated substrate by spin coating. A polymethyl methacrylate (PMMA) material is used as the photoresist in the fabrication process; Fifth, proximity lithography is performed to expose the photoresist through a photomask; Sixth, some developer is applied to remove the exposed photoresist to form the pattern on the plated substrate; Seventh, a laser is used to cut the plated substrate in order to complete the integrated packaging with a complementary metal oxide semiconductor (CMOS) sensor; Eighth, a second chromium layer is plated on the cut substrate, this layer is used as the secondary reflection prevention layer and its thickness is 75 nm; Ninth, LEDs are installed under the mask. Light can pass through the etched apertures. Given that other parts are covered by a chromium layer, they stop the incident light; Finally, the LEDs, the image sensor, and the mask are packaged into a point-source focal plane. In the MEMS focal plane, the wavelength of the MEMS point sources is determined by the dichroic filter. The MEMS point sources can be controlled by the SSPIAP controller in real-time. In the SSPIAP’s imaging mode, the LEDs can be switched off and have no effect on imaging. The MEMS light sources are installed around image sensors. Accurate positions of the MEMS light sources are calculated based on the relationships between the image sensors and the optical system. For the optical remote sensor, several image sensors are butted together into one greater image sensor [37,38]. The MEMS light sources are generally placed on the butting area.

For the third step, we built a mathematical calibration model. According to the geometrical relationships between point-light source positions and their images, the mathematical calibration equation was repeatedly solved to calculate the IOPs of the SSPIAP.

In the fourth step, a controller of the SSPIAP controls the MEMS light sources. The CMOS senses the MEMS light sources images. A centroid extraction algorithm processes input images to extract the star point position. Finally, the principal distance and the principal point can be calculated using the mathematical calibration model.

### 2.2. On-Orbit Mathematical Calibration Model

**Figure 4:**
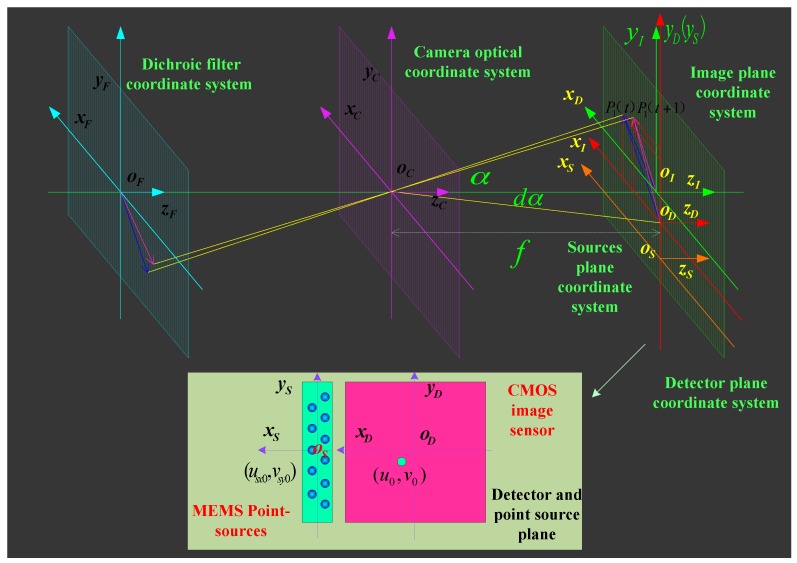
Coordinate relationship of equivalent optical path.

In [39], a coordinate transform method was used to build the relationships between ground object targets and image positions. This coordinate transform method can obtain the instantaneous imaging geometrical relationships of optical remote sensing sensors. In this study, we adopt this coordinate transform to build a mathematical model of point sources and their image positions to calculate the instantaneous principal distance and principal point. According to Figure 1, several coordinate systems from the MEMS point sources to the dichroic filter plane were defined as follows: dichroic filter plane coordinate system *F*(*x_F_*,*y_F_*,*z_F_*), camera coordinate system *C*(*x_C_*,*y_C_*,*z_C_*), image plane coordinate system *I*(*x_I_*,*y_I_*,*z_I_*), source plane coordinate system *S*(*x_S_*,*y_S_*,*z_S_*), and detector plane coordinate system *D*(*x_D_*,*y_D_*,*z_D_*). The coordinate relationships of equivalent optical paths between point sources and the image positions are shown in Figure 4.

We define $M_{t}\left(u_{0}^{t},v_{0}^{t},f_{0}^{t}\right)$ as the elements of interior orientation at time *t*. In the *S* coordinate system, the position of a point source *S*_1_ is defined as $S_{1}\left(x_{s_{1}}^{t},y_{s_{1}}^{t},0\right)$. In the *C* coordinate system, the position vector of the point source can be expressed as follows:

(1) $${\vec{n}}_{1}={\left[\begin{array}{ccc} x_{c}^{s_{1}} & y_{c}^{s_{1}} & z_{c}^{s_{1}} \end{array}\right]}^{T}=M_{3}M_{2}M_{1}{\left[x_{s_{1}}^{t},y_{s_{1}}^{t},1\right]}^{T}$$

(2) $$M_{1}=\left[\begin{array}{ccc} 1/d_{x} & 0 & u_{sx0}\\ 0 & 1/d_{y} & v_{sy0}\\ 0 & 0 & 1 \end{array}\right],M_{2}=\left[\begin{array}{ccc} d_{x} & 0 & -d_{x}u_{0}^{t}\\ 0 & d_{y} & -d_{y}v_{0}^{t}\\ 0 & 0 & 1 \end{array}\right],M_{3}=\left[\begin{array}{ccc} 1 & 0 & 0\\ 0 & 1 & 0\\ 0 & 0 & f_{0}^{t} \end{array}\right]$$

where *d_x_* and *d_y_* are the sizes of, respectively, the *x*-axis and *y*-axis detectors, and (*u_sx_*_0_,*v_sy_*_0_) is the coordinate position of the center of the MEMS point sources in the *D* coordinate system. (*u_sx_*_0_,*v_sy_*_0_) can be obtained from the design value of the focal plane of the SSPIAP. At the adjustment stage, optical collimation and precise angle measurements are used to calibrate the SSPIAP to remove preparation and adjustment error. In our calibration process, a large-scale collimator and photoelectric theodolite are used to complete calibration of the center of the CMOS and (*u_sx_*_0_,*v_sy_*_0_). From Equation (1), the unit vector of the principal ray emitted by the point source *S*_1_ in the *C* coordinate system can be expressed as follows:

(3) $${\vec{u}}_{1}={\left[\begin{array}{ccc} u_{1x} & u_{1y} & u_{1z} \end{array}\right]}^{T}=\frac{{\left[x_{s_{1}}^{t}+u_{sx_{0}}d_{x}-d_{x}u_{0}^{t},y_{s_{1}}^{t}+v_{sy_{0}}d_{y}-d_{y}v_{0}^{t},f_{0}^{t}\right]}^{T}}{\sqrt{{\left(x_{s_{1}}^{t}+u_{sx_{0}}d_{x}-d_{x}u_{0}^{t}\right)}^{2}+{\left(y_{s_{1}}^{t}+v_{sy_{0}}d_{y}-d_{y}v_{0}^{t}\right)}^{2}+{\left(f_{0}^{t}\right)}^{2}}}$$

Light emitted by the point source with position *S*_1_ passes through the camera optical system to become parallel light. The emitted parallel light from the camera’s optical system is reflected by the dichroic filter and then returned into the camera’s optical system. In the C coordinate system, the normal vector of the dichroic filter is defined as $\vec{n}={\left[n_{c_{x}}^{df}n_{c_{y}}^{df}n_{c_{z}}^{df}\right]}^{T}$. The unit vector of the principal ray of the reflected beam is defined as ${\vec{u}}_{1}^{\prime }$. When the reflected beam passes through an optical system, an image point is obtained and then received by detectors. In this case, we define the image point as $S_{1}^{\prime }$. In the *D* plane coordinate system, the position of the centroid of the image point is defined as $\left(x_{s_{1}^{\prime }}^{t},y_{s_{1}^{\prime }}^{t},0\right)$. In the *C* plane coordinate system, the position of the centroid of image point can be expressed as follows:

(4) $${\vec{n}}_{2}={\left[\begin{array}{ccc} x_{c}^{s_{1}^{\prime }} & y_{c}^{s_{1}^{\prime }} & z_{c}^{s_{1}^{\prime }} \end{array}\right]}^{T}=M_{3}M_{1}^{-1}{\left[\begin{array}{ccc} x_{s_{1}^{\prime }}^{t} & y_{s_{1}^{\prime }}^{t} & 1 \end{array}\right]}^{T}$$

(5) $$M_{1}=\left[\begin{array}{ccc} 1/d_{x} & 0 & u_{0}^{t}\\ 0 & 1/d_{y} & v_{0}^{t}\\ 0 & 0 & 1 \end{array}\right]$$

From Equation (4), the unit vector ${\vec{u}}_{1}^{\prime }$ in the *C* coordinate system can be expressed as follows:

(6) $${\vec{u}}_{1}^{\prime }={\left[\begin{array}{ccc} u_{1x}^{\prime } & u_{1y}^{\prime } & u_{1z}^{\prime } \end{array}\right]}^{T}=\frac{{\left[\begin{array}{ccc} x_{s_{1}^{\prime }}^{t}d_{x}-d_{x}u_{0}^{t} & y_{s_{1}^{\prime }}^{t}d_{y}-d_{y}v_{0}^{t} & f_{0}^{t} \end{array}\right]}^{T}}{\sqrt{{\left(x_{s_{1}^{\prime }}^{t}d_{x}-d_{x}u_{0}^{t}\right)}^{2}+{\left(y_{s_{1}^{\prime }}^{t}d_{y}-d_{y}v_{0}^{t}\right)}^{2}+{\left(f_{0}^{t}\right)}^{2}}}$$

From the law of optical reflection, the relationship among the unit vector of the principal rays can be expressed as follows:

(7) $$\begin{array}{l} {\vec{u}}_{1}\times \vec{n}=-{\vec{u}}_{1}^{\prime }\times \vec{n}\\ {\vec{u}}_{1}\cdot \vec{n}={\vec{u}}_{1}^{\prime }\cdot \vec{n} \end{array}$$

The preceding equations are expanded to a set of scalar equations as follows:

(8) $$\{\begin{array}{c} u_{1y}n_{c_{z}}^{df}-u_{1z}n_{c_{y}}^{df}=u_{1z}^{\prime }n_{c_{y}}^{df}-u_{1y}^{\prime }n_{c_{z}}^{df}\\ u_{1x}n_{c_{z}}^{df}-u_{1z}n_{c_{x}}^{df}=u_{1z}^{\prime }n_{c_{x}}^{df}-u_{1x}^{\prime }n_{c_{z}}^{df}\\ u_{1x}n_{c_{x}}^{df}+u_{1y}n_{c_{y}}^{df}+u_{1z}n_{c_{z}}^{df}=u_{1x}^{\prime }n_{c_{x}}^{df}+u_{1y}^{\prime }n_{c_{y}}^{df}+u_{1z}^{\prime }n_{c_{z}}^{df} \end{array}$$

Equations (7) and (8) represent the mathematical, spatial relationship equations between a point source and its image point. The mathematical modeling of the principal distance and the principal point can be established based on Equations (7) and (8). In our method, several conjugate pairs of MEMS point sources are symmetrically placed around the principal point.

**Figure 5:**
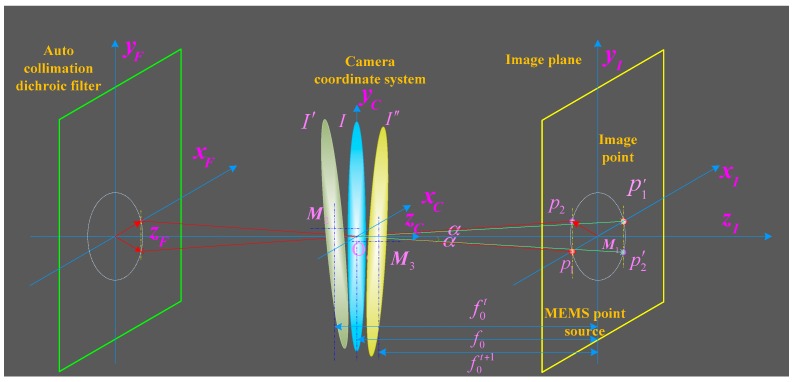
Imaging relationship in actual imaging system.

**Figure 6:**
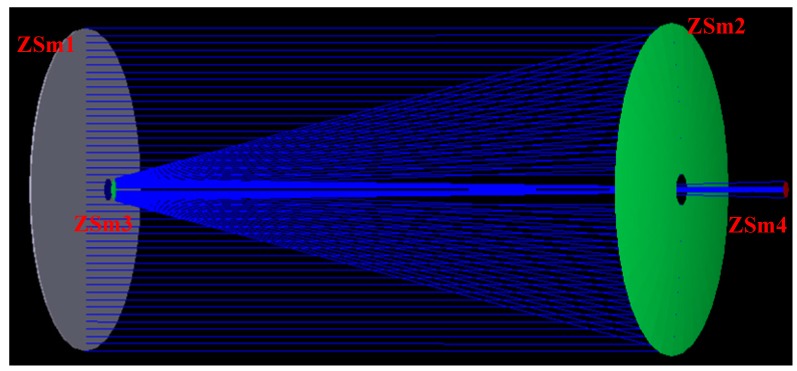
Simulation of optical system.

In an actual optical system, MEMS point sources need to be installed around the image sensor. The imaging relationship of the equivalent optical path of self-calibration is shown in Figure 5. Let *p*_1_ and *p*_2_ be two installed point sources around the image sensor. To avoid influencing the imaging, *p*_1_ and *p*_2_ are installed on the same side of the image sensor. Let the position of point sources *p*_1_ and *p*_2_ be $\left(x_{s_{1}},y_{s_{1}}\right)$ and $\left(x_{s_{2}},y_{s_{2}}\right)$ in the *S* coordinate system, respectively. Let their respective images $p_{1}^{\prime }$ and $p_{2}^{\prime }$ in the *D* coordinate system be located at $\left(x_{p_{1}},y_{p_{1}}\right)$ and $\left(x_{p_{2}},y_{p_{2}}\right)$, respectively. At the initial time, let the angles between the principal ray emitted from *p*_1_ and *p*_2_ and the optical axis be *α*_1_ and *α*_2_, respectively. Our system is designed with equal angles between the optical axis and principal rays emitted from *p*_1_ and *p*_2_. When the optical system is ideal and not maladjusted at the *I* position in Figure 6, *α*_1_ is equal to *α*_2_ (*α*_1_ = *α*_2_). The optical system is not ideal if it is affected by the manufacture and adjustment of the optical system. In an actual optical system or when maladjustment occurs (at the *I*’ and *I*” positions in Figure 6) *α*_1_ is not equal to *α*_2_ (*α*_1_ ≠ *α*_2_).

From Equations (7) and (8), the imaging relationship can be expressed as follows:

(9) $$\left\{ \begin{array}{l} f_{0}^{t}\tan({\alpha_{1}}^{t})=\sqrt{{\left(x_{s1}^{t}+u_{sx_{0}}d_{x}-d_{x}u_{0}^{t}\right)}^{2}+{\left(y_{s1}^{t}+v_{sy_{0}}d_{y}-d_{y}v_{0}^{t}\right)}^{2}}\\ f_{0}^{t}\tan(-{\alpha_{1}}^{t})=-\sqrt{{\left(x_{p1}^{t}d_{x}-d_{x}u_{0}^{t}\right)}^{2}+{\left(y_{p1}^{t}d_{y}-d_{y}v_{0}^{t}\right)}^{2}} \end{array}\right.$$

(10) $$\left\{ \begin{array}{l} f_{0}^{t}\tan({\alpha_{2}}^{t})=\sqrt{{\left(x_{s2}^{t}+u_{sx_{0}}d_{x}-d_{x}u_{0}^{t}\right)}^{2}+{\left(y_{s2}^{t}+v_{sy_{0}}d_{y}-d_{y}v_{0}^{t}\right)}^{2}}\\ f_{0}^{t}\tan(-{\alpha_{2}}^{t})=-\sqrt{{\left(x_{p2}^{t}d_{x}-d_{x}u_{0}^{t}\right)}^{2}+{\left(y_{p2}^{t}d_{y}-d_{y}v_{0}^{t}\right)}^{2}} \end{array}\right.$$

For the remote sensing optical camera, the focal length is relatively long [40,41]. The focal length generally ranges from several meters to tens meters. Variations of the principal point generally occur at the micrometer level in on-orbit working conditions. Therefore, as the specifications are expressed as follows: $\left|u_{0}^{t}-u_{0}\right|\ll \left\{f_{0}^{t},f_{0},f_{0}^{t+1}\right\}$ and $\left|v_{0}^{t}-v_{0}\right|\ll \left\{f_{0}^{t},f_{0},f_{0}^{t+1}\right\}$. The approximation of the angles between the principal rays emitted from *p*_1_ and *p*_2_ and the optical axis can be expressed as $\alpha_{1}^{t+1}\approx \alpha_{1}^{t}\approx \alpha_{1},\;\alpha_{2}^{t+1}\approx \alpha_{2}^{t}\approx \alpha_{2}$. For the optical system of the SSPIAP, the angles between the principal rays of the MEMS point sources and the optical axis are approximately equal to the designed value. From Equations (9) and (10), the following equation can be obtained:

(11) $$\left[\begin{array}{cc} a_{11} & a_{12}\\ a_{21} & a_{22} \end{array}\right]\left[\begin{array}{c} u_{0}^{t}\\ v_{0}^{t} \end{array}\right]=\left[\begin{array}{c} s_{1}\\ s_{2} \end{array}\right]$$

where $a_{11}=2d_{x}x_{s1}^{t}+2u_{sx_{0}}{d_{x}}^{2}-2{d_{x}}^{2}x_{p1}^{t}$, $a_{12}=2d_{y}y_{s1}^{t}+2v_{sx_{0}}{d_{y}}^{2}-2{d_{y}}^{2}y_{p1}^{t}$, $a_{21}=2d_{x}x_{s2}^{t}+2u_{sx_{0}}{d_{x}}^{2}-2{d_{x}}^{2}x_{p2}^{t}$, $a_{22}=2d_{y}y_{s2}^{t}+2v_{sy_{0}}{d_{y}}^{2}-2{d_{y}}^{2}y_{p2}^{t}$, $s_{1}={\left(x_{s2}^{t}+u_{sx0}d_{x}\right)}^{2}+{\left(y_{s2}^{t}+v_{sy_{0}}d_{y}\right)}^{2}-\;{\left(x_{p2}^{t}d_{x}\right)}^{2}-{\left(y_{p2}^{t}d_{y}\right)}^{2}$, $s_{2}={\left(x_{s2}^{t}+u_{sx_{0}}d_{x}\right)}^{2}+{\left(y_{s2}^{t}+v_{sy_{0}}d_{y}\right)}^{2}-{\left(x_{p2}^{t}d_{x}\right)}^{2}-{\left(y_{p2}^{t}d_{y}\right)}^{2}$.

From Equation (11), the position of the principal point can be expressed as follows:

(12) $$u_{0}^{t}=\frac{s_{1}a_{22}-s_{2}a_{12}}{a_{11}a_{22}-a_{21}a_{12}}，v_{0}^{t}=\frac{s_{1}a_{21}-s_{2}a_{11}}{a_{12}a_{21}-a_{11}a_{12}}$$

From Equations (10) and (12), the principal distance can be expressed as follows:

(13) $$f_{0}^{t}=\sqrt{\frac{\sum_{i=1}^{N}{\left(x_{p_{i}}^{t}d_{x}-d_{x}\frac{s_{1}a_{22}-s_{2}a_{12}}{a_{11}a_{22}-a_{21}a_{12}}\right)}^{2}+\sum_{i=1}^{N}{\left(y_{p_{i}}^{t}d_{y}-d_{y}\frac{s_{1}a_{21}-s_{2}a_{11}}{a_{12}a_{21}-a_{11}a_{12}}\right)}^{2}}{\tan^{2}(\alpha_{1})+\tan^{2}(\alpha_{2})}}$$

where *N* is the number of MEMS point sources. We used *N* equal to 2. In this case, the variation of the principal distance and point can be obtained as $\frac{\partial f_{0}^{t}}{\partial t}=\;f_{0}^{t+1}-f_{0}^{t},\;\;\;\;\frac{\partial u_{0}^{t}}{\partial t}=u_{0}^{t+1}-u_{0}^{t}$, and $\frac{\partial v_{0}^{t}}{\partial t}=v_{0}^{t+1}-v_{0}^{t}$. *α*_1_, *α*_2_ and the positions of two point sources can be accurately calibrated at the adjustment stage of the SSPIAP. The positions of two point source images can be calculated by the centroid extraction algorithm. Therefore, the variations of the principal distance and point can be determined.

## 3. Experiment and Analysis

### 3.1. Simulation and Analysis

To verify the effectiveness of the proposed method, we used an optical design program, ZEMAX (Zemax, LLC, Bellevue, WA, USA), to simulate calibration experiments. In ZEMAX, our input optical system model parameters were as follows: the focal length was 2032 mm and the aperture diameter was 203.2 mm. The Cassegrain optical system was adopted. The optical system was composed of a primary mirror and a secondary mirror. Based on the optical system, a monitoring optical path of the principal distance and the principal point was designed. A tertiary mirror was used in addition to the secondary mirror. The optical rays are reflected from the mirror, go through the optical system again, and are then concentrated on the CMOS detector. The designed optical path is shown in Figure 6. In this figure, ZSm1 is a mirror that simulates the dichroic filter, ZSm2 and ZSm3 are the primary mirror and secondary mirrors, respectively, and ZSm4 is a focal plane that simulates the MEMS sources.

The simulation experiment includes two steps. In the first step, the IOPs are calibrated without maladjustment in the optical system. In the second step, different maladjustments are deliberately used to calibrate the IOPs. In each step, the reference values of the principal distance and the principal point are first calculated under different maladjustments. For a maladjusted optical system, the coordinate positions from rays with different view fields to the image plane can be traced accurately. We used the on-ground calibration method based on measuring angles to calibrate the reference value of the principal distance and the principal point. The laboratory measuring angle method is widely used in the calibration of IOPs of remote sensing cameras [42,43,44]. In this method, an uncalibrated optical camera is placed on a precision turntable to capture the parallel lights of star points emitted by a collimator.

At different rotation angles of the turntable, the camera can obtain multiple positions of the star points and their images from different field of views (FOVs). The recorded rotation angle and image point positions are used to build an imaging geometrical equation between each star point and its image. From the principle of camera distortion, an optical camera has its minimum distortion at its principal point location [44,45]. A least squares method is used to solve the imaging geometrical equation to calculate the principal distance and the principal point. This method is simple and has high calibration accuracy. The calibration accuracy can reach the micrometer level [45,46]. As with the ground calibration method of the principal distance and the principal point, we used a least squares, multiple regression analysis to calculate the principal distance and the principal point of each test, maladjusted system [46,47]. The principal distance and the principal point can be expressed as follows:

(14) $$f=\frac{\left(\sum_{i=1}^{N}L_{i}\tan^{2}W_{i}\cdot \sum_{i=1}^{N}\tan^{3}W_{i}\right)-\left(\sum_{i=1}^{N}L_{i}\tan W_{i}\cdot \sum_{i=1}^{N}\tan^{4}W_{i}\right)}{{\left(\sum_{i=1}^{N}\tan^{3}W_{i}\right)}^{2}-\left(\sum_{i=1}^{N}\tan^{2}W_{i}\cdot \sum_{i=1}^{N}\tan^{4}W_{i}\right)}$$

(15) $$p_{x/y}=\frac{\left(\sum_{i=1}^{N}L_{i}\tan^{2}W_{i}\cdot \sum_{i=1}^{N}\tan^{2}W_{i}\right)-\left(\sum_{i=1}^{N}L_{i}\tan W_{i}\cdot \sum_{i=1}^{N}\tan^{3}W_{i}\right)}{{\left(\sum_{i=1}^{N}\tan^{3}W_{i}\right)}^{2}-\left(\sum_{i=1}^{N}\tan^{2}W_{i}\cdot \sum_{i=1}^{N}\tan^{4}W_{i}\right)}$$

where *f* is the principal distance, *p_x/y_* is the position of the principal point in the *x* direction or the *y* direction, *i* is the number of measurement points, *W_i_* is the measurement angle of the *i*th measurement point, and *L_i_* is the measurement height of the *i*th measurement point.

**Figure 7:**
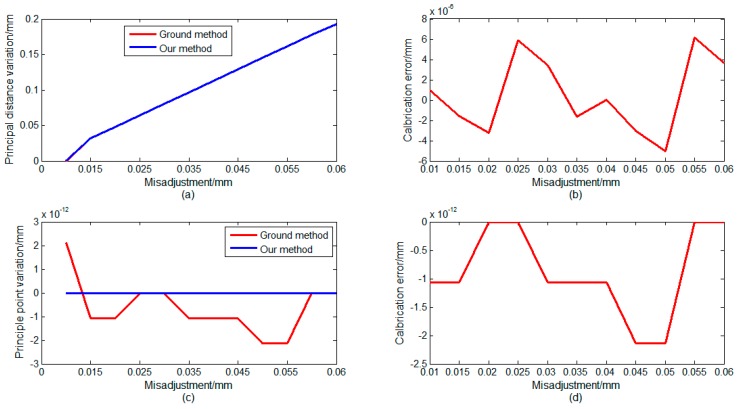
Simulation results of calibration when a mis-adjustment occurs in Z direction: (**a**) calculation results of variation of principal distance; (**b**) calibration error of two methods; (**c**) calculation results of vibration of principal point; and (**d**) calibration error of two methods.

In the first step, we used two different methods to calculate the principal distance when the optical system had no maladjustment (see the optical system in Figure 7). In our simulation, we used ten reference points at different FOV positions to estimate the principal distances and points for the on-ground method. The calculated results of the principal distance and the principal point in the simulation condition when the optical system had no maladjustments are shown in Table 1. This table presents the initial values of the principal distance and the principal point based on the two methods. For the ground method, the calculated error was 0.000853 μm. This error was produced by least squares estimation. This error can be accepted in our system. The initial values of the two methods can be considered approximately equal.

In on-orbit working conditions, the optical system may possess a variety of maladjustments. We set different maladjustments for the ZSm2. Using the same approach, we set ten reference points in a valid FOV. In the second step, we simulated three main maladjustment situations. First, we set maladjustments in the *z*-direction. We set twelve maladjustments of ZSm2 along the *z*-direction to test the proposed method. The deviations of the ZSm2 mirror from its original position were from 0.010 mm to 0.06 mm. For different maladjustment values, Figure 7 shows the calculation results using different methods. As shown in Figure 7, the principal point has not changed, but the principal distance has changed. This phenomenon is caused by the translation of the ZSm2 mirror along the optical axis direction. This case can be equivalent to a defocus phenomenon. In on-orbit working conditions, the defocus phenomenon often occurs. This case simulates the most common disorder situation. The variation of the principal distance can be accurately monitored, as shown in Figure 7. The difference between the two methods was less than 0.008 μm and this is acceptable.

**Figure 8:**
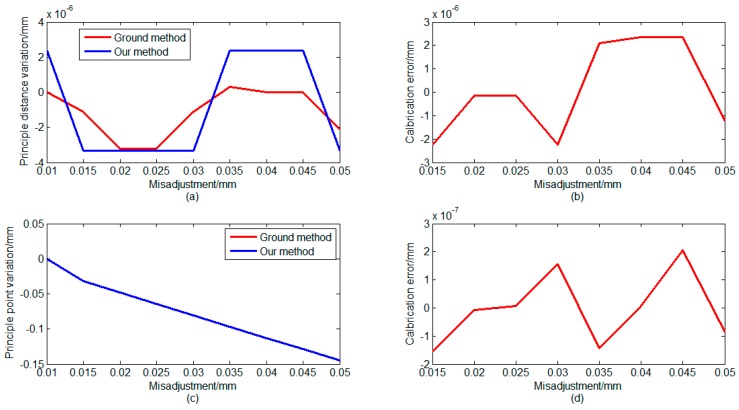
Simulation results of calibration when a misadjustment occurs in the Y direction: (**a**) calculation results of variation of principal distance; (**b**) calibration error of two methods; (**c**) calculation results of vibration of principal point; and (**d**) calibration error of two methods.

**Figure 9:**
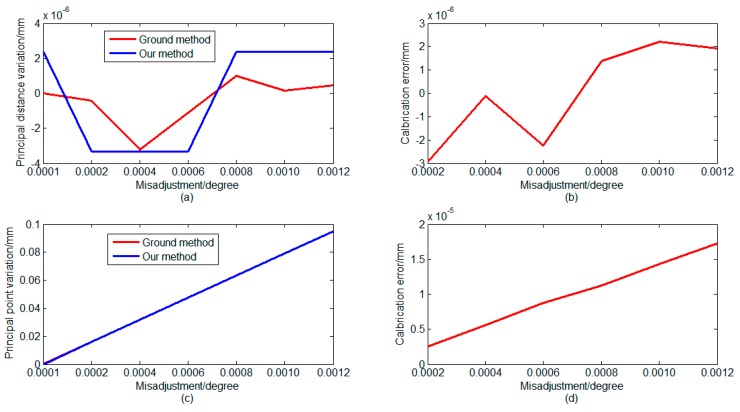
Simulation results of calibration when a misadjustment occurs around the X-axis: (**a**) calculation results of variation of principal distance (**b**); calibration error of two methods; (**c**) calculation results of vibration of principal point; and (**d**) calibration error of two methods.

Second, we set maladjustments in the *y*-direction and around the *x*-axis. In the *y*-direction, we set nine maladjustments of ZSm2 to test the proposed method. The deviations of the ZSm2 mirror from its original position were from 0.010 mm to 0.05 mm. Around *x*-axis, we set seven maladjustments of ZSm2 to test the proposed method. The deviations of the ZSm2 mirror from its original position were from 0.0001 degrees to 0.0012 degrees. Figure 8 and Figure 9 show the calculation results using different methods. Figure 8 and Figure 9 indicate that the principal distance has not changed and the principal point has changed. 

These results are due to the translation of the ZSm2 mirror away from the optical axis direction and also the inclination toward the optical axis direction. These cases can be equivalent to a mismatch between the primary mirror and secondary mirror in on-orbit working conditions. These phenomena happen during on-orbit dynamic imaging. As shown in Figure 8 and Figure 9, the error is less than 1 μm under the different maladjustments, this is acceptable. Table 1 and Figure 7, Figure 8 and Figure 9 show that our method can accurately calibrate the variation of the principal distance of the principal point. In Figure 7, Figure 8 and Figure 9, the calibration error is the difference between the two methods. In Figure 9, the calibration error shows a linear growth because the larger maladjustments make larger deformations of the optical system and the images. Compared with the error in the on-ground method, the error in our method is less than 1 μm.

### 3.2. Experiments

**Figure 10:**
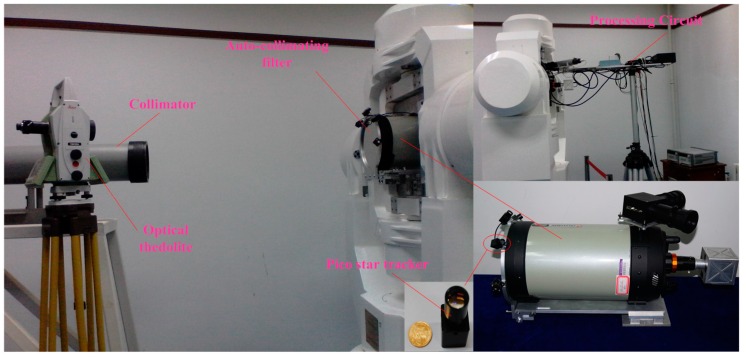
Experimental system.

We set up an experimental system for integrated calibration with imaging to verify the proposed method. Figure 10 shows the experimental calibration system.

The system included an optical camera, an auto-collimating filter, a processing circuit, a collimator, an optical theodolite, and a high-accuracy turntable. The optical system was a co-axial Schmidt–Cassegrain optical system (Celestron, Torrance, CA, USA). The designed value of the aperture diameter was 203.2 mm, and that for the focal length was 2032 mm, and the F/ratio of the optical system was 10. The image sensor was a CMOS detector, and the image resolution was 1280 × 1024 pixels. The MEMS point sources and image sensor were installed on the focal plane. A collimator provided an infinite target for the test system.

**Figure 11:**
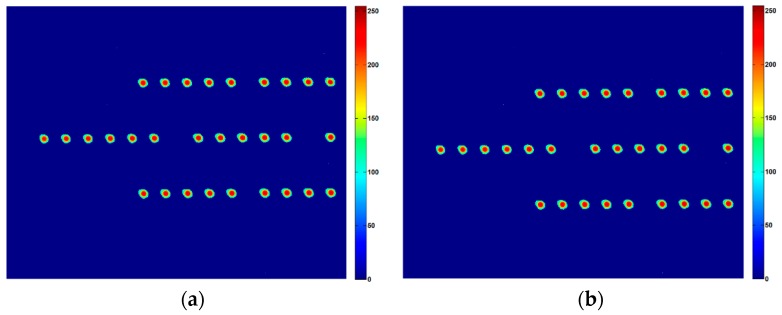

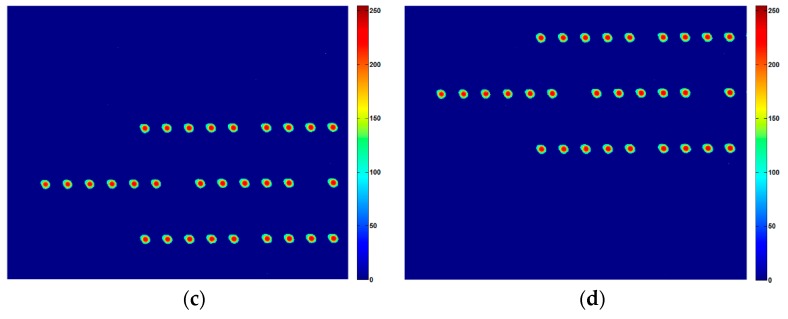
Image at different angles. (**a**) is 0 degree; (**b**) is −0.0225 degrees; (**c**) is −0.04 degrees; (**d**) is +0.04 degrees.

Firstly, we calibrated the reference value of the principal distance and principal point. The camera controller set the imaging mode. The three-axis turntable was adjusted evenly using the level. The collimator was also adjusted evenly. By adjusting the support tooling of the camera, and using the benchmark prisms of the camera’s optical axis, the camera’s visual axis and collimator were moved to share a common shaft. Star points of the collimator were imaged on the target CMOS sensor. The turntable was revolved, and the rotation angle was recorded. The captured image was also recorded. The processing circuit output star coordinates in real-time. Figure 11 shows the captured images.

**Figure 12:**
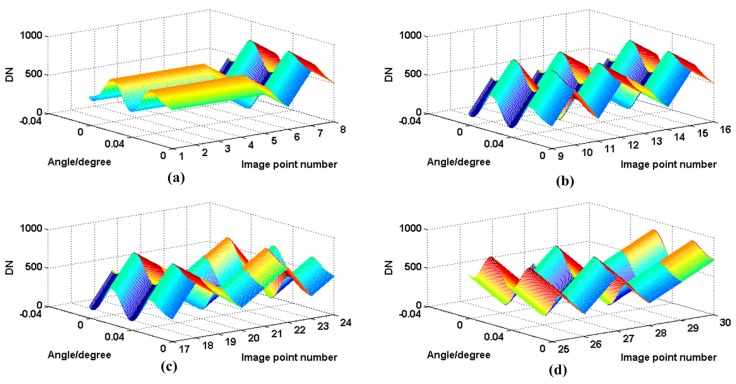
Centroid position of different points. (**a**) is the image points 1–8; (**b**) is the image points 9–16; (**c**) is the image points 17–24; (**d**) is image points 25–30.

To avoid startup and pause vibration errors, the turntable was rotated at the same period. Images and rotation angles were recorded in real-time. Figure 12 shows the centroid positions in a period.

Based on the measured centroid position and rotation angle, least squares, two-multiplication regression analysis was used to obtain the optimal estimation values of the IOPs. Table 2 shows the calculation results.

**Figure 13:**
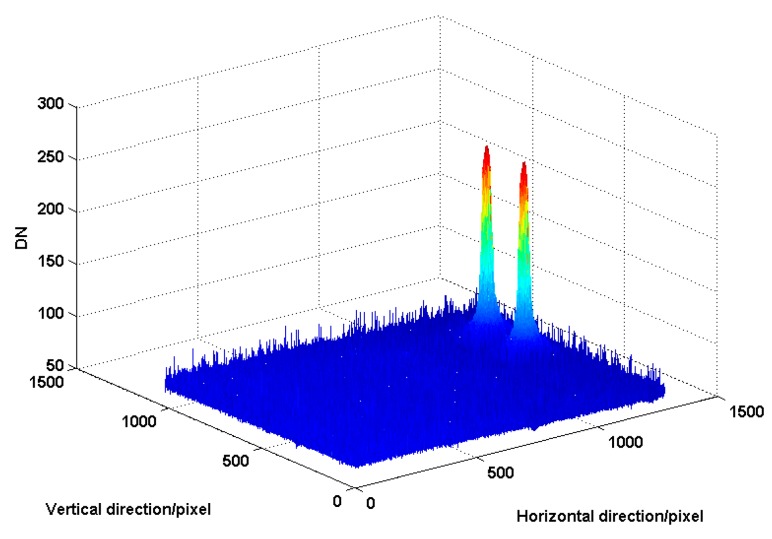
Gray distribution of captured image for our method.

Secondly, we calibrated the principal distance and the principal point using our method. In the SSPIAP, the camera controller set the calibration mode and switched the MEMS point sources on or off. The MEMS point sources were lit when the camera controller executed the turn-on command. Figure 13 shows the captured images when two point sources were lit. Using the spot centroid algorithm, the position of images can be determined. The principal distance and the principal point were calculated according to Equations (12) and (13).

Table 3 shows the calculation results. The principal distance calibrated by our method was 2032.0818 mm and the standard deviation was 0.0343 mm. The deviation is a constant error. The error was mainly be produced by the on-ground method error, calibration errors of positions (*x_s_*_1_,*y_s_*_1_) and (*x_s_*_2_,*y_s_*_2_), centroid extraction error, and error in the installation of the focal plane. For the on-ground method, the calibration accuracy was better than 5 μm [44,45]. The position accuracy of the spot image is mainly determined by the centroid extraction algorithm. For the centroid extraction algorithm, the measurement accuracy can reach 0.05 pixels. The pixel size of the image sensor is 5.3 μm in the SSPIAP system. Thus, the extraction accuracy can reach 0.265 μm. The total standard deviation of the principal distance and the principal point can be expressed as follows:

(16) $$\sigma_{f_{0}^{t}}=\sqrt{\sum_{i=1}^{N}{\left(\frac{\partial f_{0}^{t}}{\partial x_{p_{i}}^{t}}\right)}^{2}\sigma_{x_{p}}^{2}+\sum_{i=1}^{N}{\left(\frac{\partial f_{0}^{t}}{\partial y_{p_{i}}^{t}}\right)}^{2}\sigma_{y_{p}}^{2}}$$

(17) $$\sigma_{u_{0}^{t}}=\sqrt{\sum_{i=1}^{N}{\left(\frac{\partial u_{0}^{t}}{\partial x_{p_{i}}^{t}}\right)}^{2}\sigma_{x_{p}}^{2}+\sum_{i=1}^{N}{\left(\frac{\partial u_{0}^{t}}{\partial y_{p_{i}}^{t}}\right)}^{2}\sigma_{y_{p}}^{2}}$$

(18) $$\sigma_{v_{0}^{t}}=\sqrt{\sum_{i=1}^{N}{\left(\frac{\partial v_{0}^{t}}{\partial x_{p_{i}}^{t}}\right)}^{2}\sigma_{x_{p}}^{2}+\sum_{i=1}^{N}{\left(\frac{\partial v_{0}^{t}}{\partial y_{p_{i}}^{t}}\right)}^{2}\sigma_{y_{p}}^{2}}$$

where *N* is the number of point sources, and $\sigma_{x_{p}}$ and $\sigma_{y_{p}}$are the measurement accuracies of the centroid extraction algorithm in the *x* and *y* directions. That is, $\sigma_{x_{p}}$ = $\sigma_{y_{p}}$ = 0.265 μm. Based on Equations (16) and (18), the total standard deviation of the principal distance and the principal point is less than 0.02 μm. Therefore, the centroid extraction error was relatively small and can be neglected. The calibration errors of positions (*x_s_*_1_,*y_s_*_1_) and (*x_s_*_2_,*y_s_*_2_) and the installation errors of the focal plane were controlled to be less than 30 μm. However, these errors do not affect the monitoring of the relative variation of the principal distance and the principal point.

**Figure 14:**
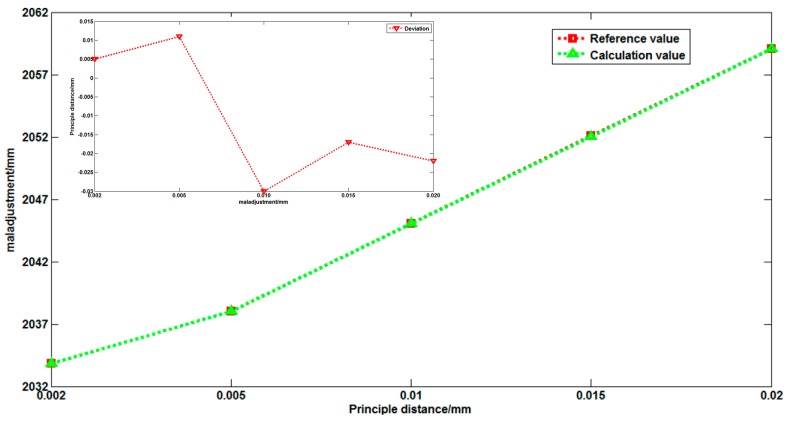
Calibration testing under maladjusted condition.

Thirdly, we adjusted the motion of the secondary mirror to simulate on-orbit maladjustments of the optical system. From analyzing the maladjustments of different elements of the optical system, we know that the secondary mirror and primary mirror maladjustments have an effect on the focal plane of the optical system. Given that the primary mirror is installed in the primary mirror room, it has almost no maladjustment. Figure 14 shows the principal distance variation under different maladjustments. In addition, we used the ground calibration method based on a least squares, multiple regression analysis as reference. The calibration deviation was less than 0.015 mm. The deviation stems from the self-method error between methods under the maladjusted condition.

In the experimental setup, the monitoring accuracy of the variation of the principal distance and the principal point were mainly affected by turntable vibrations, environmental vibrations, temperature, and airflows.

**Figure 15:**
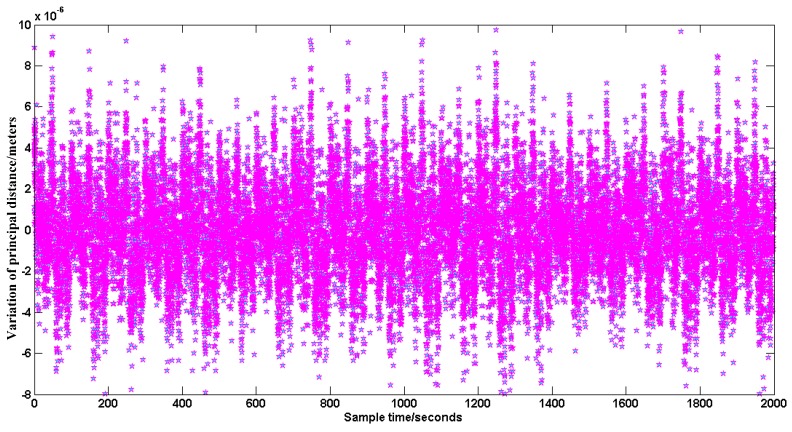
Variation of principal distance.

**Figure 16:**
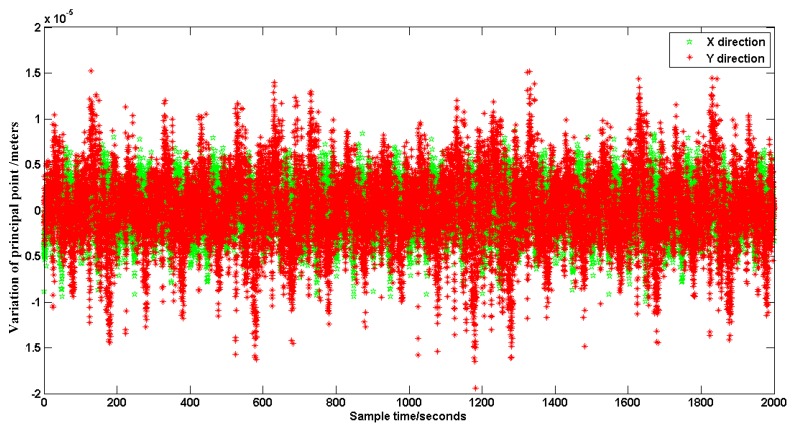
Variation of principal point.

When the SSPIAP is working in a space environment, the centroid extraction and platform flutter temperature are the primary error sources. In the SSPIAP system, we designed a special thermal control system to maintain minimal changes in the temperature field of the optical system. Further, we use a manganin material in a structure designed for vibration attenuation in the SSPIAP. The vibration attenuation structure can significantly reduce the influence of flutter on the optical system. The centroid extraction error mainly depends on the centroid extraction algorithm. The extraction accuracy of our method is less than 0.05 pixels. In our SSPIAP system, the pixel size of the image sensor is at the micrometer level. Thus, the extraction accuracy is less than 1 μm, and the error is acceptable for the requirement of errors less than 50 μm. To test the monitoring accuracy of our method in an approximation to the on-orbit environment, our experiment was performed in a laboratory with constant temperature. Furthermore, the experimental turntable used a gas-floating vibration isolation platform to avoid vibration disturbance. We used our method to monitor the variation of the principal distance and the principal point in the static case. We processed tens of thousands of images to calculate the monitoring accuracy. The variation of the principal distance in 2000 real-time seconds is shown in Figure 15, while the corresponding variation of the principal point position is presented in Figure 16.

Based on the statistical data in Figure 15 and Figure 16, the mean square error formula is used to calculate the monitoring accuracy. The monitoring accuracy of the principal distance can reach 2.4 μm. The monitoring accuracy of the principal point can reach 2.6 μm and 3.9 μm in the *x* and *y* directions, respectively. The SSPIAP has 5 m image positioning accuracy. It requires less than 50 μm calibration accuracy of the principal distance and the principal point. The monitoring accuracy can reach the micrometer level and meet the SSPIAP mapping requirements.

## 4. Conclusions

In this paper, we discussed a high-accuracy on-orbit calibration method or the IOPs of an SSPIAP. We adopted an integrated method to build an auto-collimation self-calibration system. An auto-collimation dichroic filter and MEMS point sources were integrated into the SSPIAP. First, the point sources were installed on the focal plane, and we used the MEMS method to fabricate point sources and package them with the image sensor; Second, we integrated the auto-collimation dichroic filter into the optical systems of the SSPIAP; Third, a mathematical model of IOPs was built based on a geometrical imaging model; Fourth, the centroid extraction algorithm was used to process images to extract the star point position to calculate the IOPs; Finally, we used ZEMAX to simulate the proposed method and set up an experiment to verify the feasibility of our method. The monitoring accuracy can reach micrometer levels. The proposed method can complete self-calibration without space and time limitations in real-time. In addition, our method can be applied to other calibration methods to improve their performance.

## Figures and Tables

**Table 1:** Simulation calculation results without maladjustments.

Elements	Ground Method	Our Method	Misadjustment (mm)
f (mm)	2031.999999	2032.000	0
Δf (mm)	8.527259 × 10^−7^	0	0
U0x (mm)	0	0	0
U0y (mm)	0	0	0
ΔU0x (mm)	0	0	0
ΔU0y (mm)	0	0	0

**Table 2:** Calculation results of principal distance and principal point using ground methods.

Number	Elements	Reference Value
1	f (mm)	2032.1161
2	U0x (mm)	–0.5856
3	U0y (mm)	–0.9643

**Table 3:** Calculation results of principal distance and principal point using our method.

Number	Elements	Calibration Value
**1**	f (mm)	2032.0818
**2**	U0x (mm)	–0.5387
**3**	U0y (mm)	–0.9580
**4**	Δf (mm)	0.0342
**5**	ΔU0x (mm)	0.0469
**6**	ΔU0y (mm)	0.0063
